# 

**Published:** 1881-10

**Authors:** 


### CHICAGO MEDICAL Journal and Examiner

EDITORS:

N. S. DAVIS, M.D., LL.D. JAS. NEVINS HYDE, A.M., M.D.

DANIEL R. BROWER, A.M. M.D.

CHICAGO MEDICAL PRESS ASSOCIATION. AUG. 2D 1875.

CHICAGO:

*PUBLISHED BY THE CHICAGO MEDICAL PRESS ASSOCIATION,*

188 Clark Street.

☞ **Read the Notice of this Journal among Advertisements.**

